# Assessing kidney development and disease using kidney organoids and CRISPR engineering

**DOI:** 10.3389/fcell.2022.948395

**Published:** 2022-09-02

**Authors:** Wajima Safi, Andrés Marco, Daniel Moya, Patricia Prado, Elena Garreta, Nuria Montserrat

**Affiliations:** ^1^ Pluripotency for Organ Regeneration. Institute for Bioengineering of Catalonia (IBEC), The Barcelona Institute of Technology (BIST), Barcelona, Spain; ^2^ Centro de Investigación Biomédica en Red en Bioingeniería, Biomateriales y Nanomedicina, Madrid, Spain; ^3^ Catalan Institution for Research and Advanced Studies (ICREA), Barcelona, Spain

**Keywords:** pluripotent stem cells, CRISPR, nephrogenesis, kidney engineering, kidney organoids

## Abstract

The differentiation of human pluripotent stem cells (hPSCs) towards organoids is one of the biggest scientific advances in regenerative medicine. Kidney organoids have not only laid the groundwork for various organ-like tissue systems but also provided insights into kidney embryonic development. Thus, several protocols for the differentiation of renal progenitors or mature cell types have been established. Insights into the interplay of developmental pathways in nephrogenesis and determination of different cell fates have enabled the *in vitro* recapitulation of nephrogenesis. Here we first provide an overview of kidney morphogenesis and patterning in the mouse model in order to dissect signalling pathways that are key to define culture conditions sustaining renal differentiation from hPSCs. Secondly, we also highlight how genome editing approaches have provided insights on the specific role of different genes and molecular pathways during renal differentiation from hPSCs. Based on this knowledge we further review how CRISPR/Cas9 technology has enabled the recapitulation and correction of cellular phenotypes associated with human renal disease. Last, we also revise how the field has positively benefited from emerging technologies as single cell RNA sequencing and discuss current limitations on kidney organoid technology that will take advantage from bioengineering solutions to help standardizing the use of this model systems to study kidney development and disease.

## 1 Introduction

Kidney diseases are world-wide a leading cause for mortality and health care costs. Chronic kidney disease (CKD) -defined as the chronic decline of glomerular filtration rate-is a risk factor for premature death (Webster et al., 2017) There are rarely therapies for underlying causes of CKD and it’s end stage renal failure (ESRD) can only be cured by renal replacement therapy (RRT) or transplantation (Wouters et al., 2015). However, long-term RRT itself poses a health risk while access to kidney transplantation is severely restricted because of the significant shortage of kidney donors. Thus, there is an urgent need for improved public awareness, prevention strategies, early detection, education, and development of therapies for CKD in clinical practice (Zoccali et al., 2018).

However, modelling kidney (patho)- physiology and function in the animal model had been hampered for a long time by the fact that most *in vivo* models do not fully and reliably recapitulate the human kidney (Kumar et al., 2014). Also, *in vitro* models lack reliability and feasibility because primary cells from patients with a lot of common renal diseases are difficult to obtain and culture long-term. Kidney-derived cell lines don’t display all characteristics of their original cell or maintain specific features only a short amount of time and therefore provide limited insights of an organ consisting of 26 distinct cells with various functions from pH regulation, excretion, erythropoiesis or blood pressure regulation (Baer et al., 1999; Al-Awqati and Oliver, 2002; Jenkinson et al., 2012). Therefore, the possibility to direct human pluripotent stem cells (hPSCs) towards a renal lineage opens exciting opportunities to develop new *in vitro* models of the kidney.

The mammalian kidney, the metanephros, is formed of complex epithelial tubules, the nephrons. Each single nephron connects with the collecting duct network through which the urinary filtrate passes to exit the kidney and move to the bladder. In humans, each of our kidneys consists of up to 2 million nephrons (Bertram et al., 2011), whereas in the mouse, this number is around 15,000 per each organ (Merlet-Benichou et al., 1999). As in humans nephron formation is exhausted by birth a lower number in nephrons due to hypertension or smoking during pregnancy has been shown to be associated with a later risk to develop CKD (Hinchliffe et al., 1991). Hence, there is no postnatal stem cell able to replace or generate *de novo* lost nephrons in humans.

Interestingly, it has been described that the adults of simple vertebrates like elasmobranch (Elger et al., 2003) and teleost fishes (Reimschuessel and Williams, 1995), as well as amphibians (Gray, 1930) and reptiles (Solomon, 1985) have the ability to regenerate entire nephrons but this ability seems to have been reduced to only the repair of nephron parts in adult mammals during the evolution (Romagnani et al., 2013). Due to the existence of such regenerative potential across the animal phylia, it has been postulated that the possibility to emulate such mechanisms in the mammalian kidney would represent an alternative strategy to promote kidney regeneration with no need of cell replacement therapies. In this regard, the regeneration after renal injury in different experimental animal models, and even humans, has been of great interest in the field (Ho et al., 2008; Shi et al., 2008; Soufi et al., 2012).

Potentially replacing damaged cells by nephron progenitors has emerged as an alternative strategy in regenerative medicine. Nephron progenitor cells (NPCs) can be isolated from the mouse embryonic kidney and further identification of cell culture conditions for their expansion *in vitro* raises the prospect of nephrogenesis *in vitro* (Brown et al., 2015). Nevertheless, access to NPCs from human embryonic kidneys is not affordable and raises important ethical concerns. An alternative approach lies in the identification of human cell sources with renal differentiation potential. Although for a long time not clear if a hierarchical relationship between NPC and mature renal cells do exist, this strategy laid the groundwork for the successful *in vitro* recapitulation of nephrogenesis. In this regard, hPSCs due to their capacity to differentiate into all three embryonic germ layers represent a unique cell source to virtually generate any cell type of our body. Profiting from this characteristic both human embryonic stem cells (hESCs) (Thomson et al., 1998) and human induced pluripotent stem cells (hiPSCs) (Takahashi et al., 2007) have been used to mimic early steps of human tissue specification and differentiation. This has led to the establishment of cell culture procedures which recapitulate developmental pathways by culturing hPSCs in cell culture media containing soluble factors or chemical compounds emulating the biochemical signalling that hPSCs encounter during tissue development. Initial approaches have largely been relying on the use of other external stimuli as extracellular matrix proteins promoting differentiation or the use of supportive cells as producers of paracrine signalling. However, this turned out to be neither cost effective nor feasible regarding prospectives of regenerative medicine like developing cell-based therapies or patient-derived tissues to regenerate a damaged organ.

In general, the hPSCs field has profited from the extensive studies of mouse kidney development and mouse hPSCs differentiation in 2D conditions. These approaches were also performed through the generation of hPSCs aggregate-like structures named embryoid bodies (EBs). For instance, this knowledge was key for the establishment of the first renal-like cells using mouse embryonic stem cells (Kim and Dressler, 2005; Bruce et al., 2007; Vigneau et al., 2007; Morizane et al., 2013; Taguchi et al., 2014). Later, other approaches did take advantage of 2D monolayer cultures from both human and mouse PSCs to derive renal progenitor cells that showed renal differentiation potential *in vivo* or *ex vivo* (Song et al., 2012; Narayanan et al., 2013). Building upon these findings, the work of different research groups, including Izpisúa Belmonte (Xia et al., 2013), Bonventre (Morizane et al., 2015) or Little (Takasato et al., 2015), proved the possibility to generate three dimensional (3D) cultures that mimic the embryonic kidney in terms of cellular composition and a wide variety of cell identities capable to functionally respond to external insults. In this context, a recent study has revealed that kidney organoids can faithfully model renal intrinsic repair upon toxin administration as well as a transition to an incomplete repair upon repetitive and a more aggressive insult, which mimics an *in vivo* kidney’s capacity faithfully (Gupta et al., 2022). Later on, the use of microfluidic platforms has allowed for the differentiation with generation of single renal cell types (Musah et al., 2017) or the vascularization of kidney organoids (Homan et al., 2019).

A more recent advance in the field is the combination of organoid technology with gene editing. This approach enables the generation of patient-derived hPSCs or genetically manipulated hPSCs carrying a disease-related mutation to further explore how genetic alterations impact kidney organoid phenotypes and function. Recent findings show that combining both kidney organoids and CRISPR/Cas9 enable the recapitulation of important physiopathological mechanisms such as cyst formation in renal polycystic disease (Xia et al., 2013; Freedman et al., 2015) or identification of the central role of podocyte microvilli in kidney dysfunction (Kim et al., 2017).

Based on these findings this review aims to provide a comprehensive overview on the latest advances in kidney organoid derivation to further discuss how the application of genome editing in these model systems is opening new possibilities to model kidney disease. We first provide an overview of the establishment of the first protocols for kidney organoid derivation. Next, we revise the different works using CRISPR/Cas9 technology to model renal disease and finally we discuss the utility of genome editing in the field of kidney organoid engineering to understand kidney development and disease. We will also provide an outlook on how the application of bioengineering is expected to improve our understanding of controlling and guiding kidney organoid maturation and function.

## 2 Kidney development in mammals

The adult kidney is composed of specialized cell types, including epithelial, endothelial, and stromal components. Both the collecting duct system and the nephron epithelium of the kidney represent the major epithelial component of this organ. These cell types emerge soon during development from a common lineage, the intermediate mesoderm (IM) which derives from the primitive streak (PS). The IM appears soon after gastrulation, in humans, by embryonic day 22 (E8.0 in mice) and is so-called due to its specific location along the mediolateral axis of the embryo between the axial (or somitic mesoderm) and the lateral plate mesoderm (Little and McMahon, 2012).

During embryonic kidney development, two different progenitor populations are derived from the IM: the ureteric bud (UB) and the metanephric mesenchyme (MM) (Little et al., 2007). In mice, the IM patterns into anterior-posterior axis from embryonic day (E) 8.5 to E9.5. At this stage, the cells from the UB lineage are derived from the anterior part of the IM (at 8.5E), whereas the MM develops from the posterior IM (at E9.5). Later on, a subset of the MM condenses around each UB tip to form the cap mesenchyme (CM), which refers to the multipotent population of progenitor cells that will form the nephrons (the filtering units of the mature kidney). From the UB the collecting duct system, which includes the collecting duct, the renal pelvis, ureter, and bladder trigone, will form. Interestingly, the pioneering work from Grobstein showed that the formation of nephrons requires a primary induction event from the UB to the CM (Grobstein, 1953; Auerbach and Grobstein, 1958). This inductive signal triggers mesenchymal to epithelial transition (MET) within the CM generating the renal vesicles (RV). The proximal portion of the RV elongates forming a comma-shaped body while distal region fuses with the adjacent ureteric tip generating a renal connecting tubule. The comma-shaped body undergoes further morphological changes to acquire an S shape. The S-shaped (SS) nephron is a transitional nephron stage with additional level of patterning which includes proximal, medial, and distal segments. Both, the distal and medial segments of the S-shaped body give rise to the epithelial tubule which is finally compartmentalized into the proximal tubule, the Loop of Henle, and the distal tubule. The proximal segment of the S-shaped body give rise to both parietal (Bowman’s capsule) and visceral (podocyte) epithelial layers that, upon invasion of endothelial and mesangial cells, generates the mature glomerulus. This cell population expresses a subset of transcription factors, such as *SIX2, PAX2*, and *Sall1* that are essential for both, maintenance of their multipotent progenitor fate and organization of their later differentiation (Torres et al., 1995; Nishinakamura et al., 2001; Osafune et al., 2006; Kobayashi et al., 2008).

Another type of precursor cell present in the MM are the stromal progenitor cells. These arise surrounding the UB tips and developing nephrons. This cell population is critical for the regulation of the NPCs and UB development. Together with NPCs, stromal cells secrete glial-cell-derived neurotrophic factor (GDNF) in the MM to promote UB branching (Magella et al., 2018). Additionally, stromal cells produce retinoic acid which in turn upregulates expression of RET in the UB and therefore contribute reciprocally to UB branching (Rosselot et al., 2010). Another function of stromal cells during kidney embryonic development is to control NPCs expansion via *FAT4* protocadherin expression which binds to *DCHS1/2* cadherin-related protein in MM to restrict progenitor self-renewal (Bagherie-Lachidan et al., 2015; Mao et al., 2015). Finally, stromal progenitor cells give rise to all the cell types that comprise the mural cell layer of renal blood and lymphoid vessels along with other relevant cell types such as glomerular mesangial cells or pericytes. The renal interstitium consists of all these cells. It`s function, apart from providing structural support to kidney through extracellular matrix (ECM) production, is to fulfill important endocrine functions with the identification of interstitial renin- and erythropoietin-producing cells (Zeisberg and Kalluri, 2015). All these findings identify NPCs, stromal progenitor cells and UB cells, as the essential precursors that develop into functional components of the mammalian kidney. Physical and chemical crosstalk between these cell lineages is critical for appropriate kidney development and homeostasis. Thus, isolation and expansion of these precursor cells and their derived mature cells is crucial to stablish *in vitro* models of kidney development, physiology, and disease.

## 3 Generating kidney organoids from hPSCs: Milestones and challenges

### 3.1 Major milestones in the field of kidney organoid derivation

Findings on the genetic mechanisms and cell processes that shape kidney development in animal models such as chick and mouse have been crucial when providing a correct understanding of early kidney embryogenesis in humans.

Recently, the field has been able to investigate human embryonic kidney samples at different stages of development. These pioneering works shed light on differences between human and mouse kidneys (Lindström et al., 2018b). With the advance of powerful tools such as single-cell RNA-sequencing it is now possible to perform a thorough characterization of the identity of the multiple cell types encountered in the human kidney as well as the proportion of a specific cell population with respect to the others. When generating this data sets from mouse and kidney organs at different stages during embryonic development the resolution of differentiation state and spatial distribution at single cell level may help to improve our understanding on kidney development. To date different reports have defined culture conditions for the isolation and expansion of embryonic kidney cells (reviewed in (Shankland et al., 2007; Romagnani and Anders, 2013; Romagnani et al., 2013).

**FIGURE 1:**
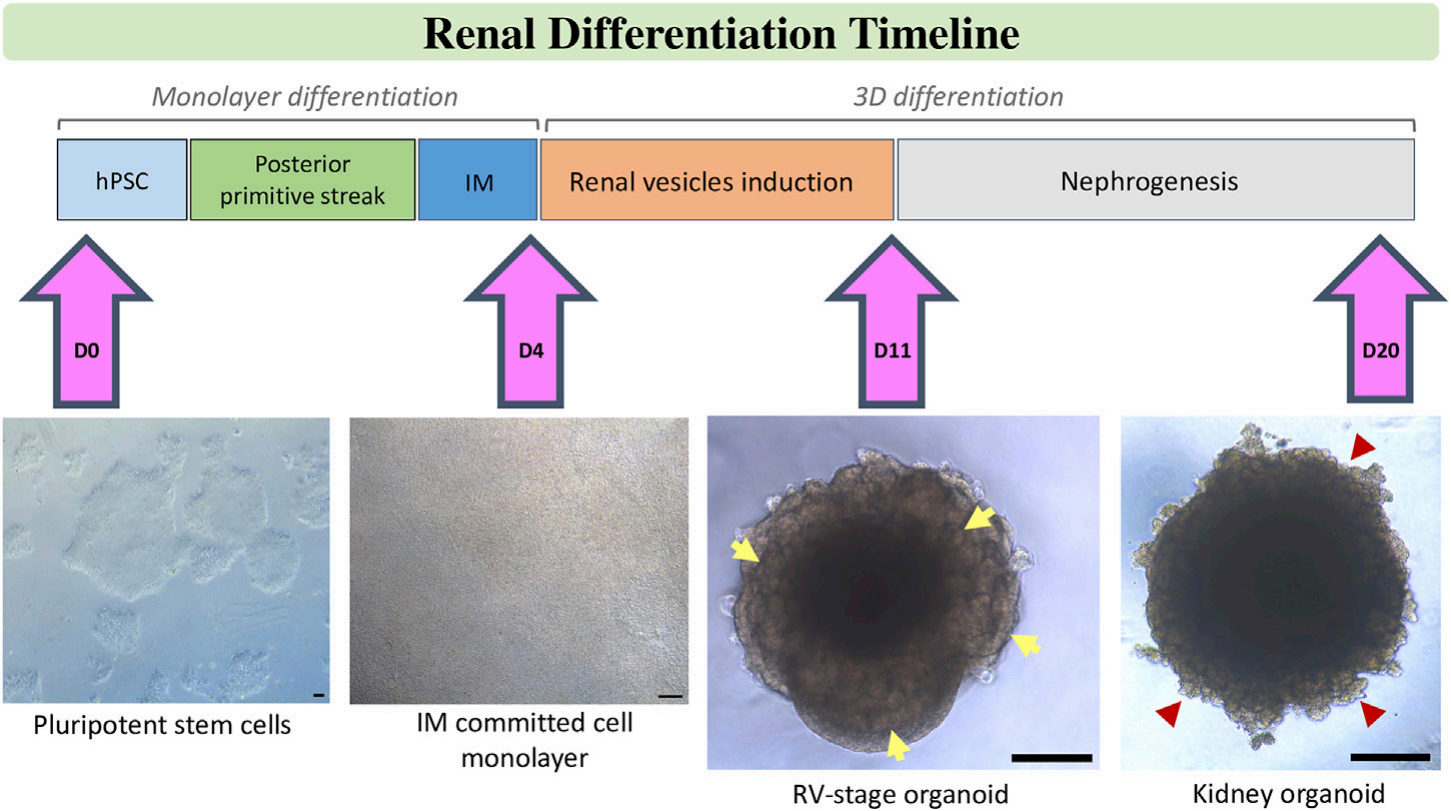
Schematic stepwise description of the differentiation of kidney organoids from hPSCs. After treatment of hPSCs with factors and small molecules according to chosen protocol posterior primitive streak is induced. Further treatment with renal inductive signals leads to intermediate mesoderm (IM) commitment. Specifically, in our protocol, this step is characterized by the formation of a tight monolayer of IM committed cells on Day 4 of differentiation. IM-comitted cells are treated with a short pulse of CHIR, then aggregated to spheroids and further stimulated with FGF9 signalling until Day 11 to induce the formation of renal vesicels (RVs), which are visible at the borders of the organoid (yellow arrows). Upon removal of factors, RVs develop into tubular and glomerular structures (red arrow heads) to generate kidney organoids by Day 20 of differentiation. Scale bars, 100 µm.

An alternative strategy for the generation of unlimited quantities of kidney-related cell types is the differentiation of hPSCs. In the last few years independent research groups have described the possibility of generating different kidney populations from hPSCs. Several procedures have been published using different sources of techniques of differentiation of hPSCs to renal cell types. They all have in common the idea to break down organogenesis to different decision points which pattern the kidney within an embryo. Insights in kidney development provide the ground to reach a desired renal cell by forcing the phenotype-specific gene expression profiles chronologically on it. Nephrogenesis, when translated to *in vitro* studies to differentiate hPSCs towards the renal lineage, has been to date divided in three consecutive steps: the induction of IM-like cells, the specification of a transitory MM giving rise to the formation of RV structures, and finally nephron formation in the absence of instructive morphogens/cytokines proving the autonomous responses of these culture model systems (Figure 1).

The aim of initial studies was to differentiate towards mature renal cell types because mature cells of the adult kidney are well characterized and the primary target of many kidney diseases. In this regard, Song and colleagues were the first ones to describe the derivation of podocyte progenitor cells from hiPSCs. In their work the authors showed that hiPSCS-derived podocytes were efficiently integrated into mouse metanephric tissues (Song et al., 2012). Other protocols on the development of renal cell types were also described by Narayanan and colleagues who described the generation of renal epithelial cells from hESCs. The differentiated stem cells exhibited markers characteristic of renal proximal tubular cells and their precursors generating tubular structures *in vitro* and *in vivo* (Narayanan et al., 2013). However, these studies didn’t yield efficiently cells of the renal lineage that could be used for further applications. Still, these studies were among the first ones to apply and establish a validation scheme of kidney development markers which proved that recapitulation of *in vivo* nephrogenesis *in vitro* might be possible by treatment of hSPCs with growth factors.

Soon after those first two studies the field started to explore the possibility to derive renal progenitor cells from hPSCs as a new approach to understand common and divergent developmental processes guiding kidney development between mice and human species. Furthermore, they aimed to generate cell sources with the potential to model these processes in the human background. In an early study Mae and colleagues tried for the first time to differentiate monolayers of hESCs towards IM using a reporter hiPSC line in which green fluorescent protein (GFP) was targeted into Odd-skipped related 1 (*OSR-1*, a gene transiently expressed in the IM during mouse embryogenesis). With this approach the authors showed that using both EB and monolayer culture methods, a sequence of growth factor combinations as GSK-3β inhibitor CHIR99021 (CHIR), Activin A, and BMP7 in the presence of fetal bovine serum led to PS formation and the generation of an OSR1^+^ IM population characterized for expression of kidney developmental markers, including *PAX2, WT1, OSR1, EYA1, LHX1*, and *CD24* over 8 days of differentiation (Mae et al., 2013).

Later studies by Bonventre and Little laboratories generated PS and IM relying on Wnt signalling to generate PS and highlighting the role of fibroblast growth factor (FGF) signalling to induce the formation of IM and MMcell populations from hPSCs, respectively (Lam et al., 2014; Takasato et al., 2014).

By the same time, the Izpisua Belmonte laboratory demonstrated the possibility to generate, for the first time, UB progenitors from both hESCs and from hiPSCs derived from patients affected by polycystic kidney disease (PKD). Making use of a two-step protocol the authors first induced mesodermal specification by bone morphogenetic protein 4 (BMP4) and FGF2. Then IM anteriorization was induced exposing the cells to retinoic acid, activin A and BMP2. After only 4 days in culture UB progenitor cells expressed *HOXB7*, *RET* and *GFRA1*. Moreover, when UB-like-hiPSC-derived cells were co-cultured with dissociated E11.5 mouse metanephric cells, UB-like-hiPSC-derived cells only integrated into cytokeratin 8 positive (+) UB-like structures, suggesting, for the first time, the induction of UB lineage-committed IM cells *ex vivo* (Xia et al., 2013). In the same manner, Taguchi and colleagues reported on the derivation of both mouse ESCs- and hiPSCs-which showed the ability to reconstitute 3D nephron-like structures (including both glomerulus- and renal tubule-like structures *in vitro*). These co-cultures made use of embryonic spinal cords as an external inducing source for kidney differentiation *ex vivo* (Taguchi et al., 2014). In regard to functional usage Imberti and colleagues demonstrated for the first time that hiPSC-derived renal progenitors robustly engrafted into damaged tubuli restoring renal function (Imberti et al., 2015). In their work, the authors generated NPCs using retinoic acid, RhoA and PI3K inhibitors and activin A to induce IM generation. IM-committed hiPSCs were treated with FGF2, BMP7 and GDNF for 13 additional days to generate MM-derived hiPSCs. Along this same line, another work from the same laboratory, showed that kidney stem cells derived from hPSC can be induced to form spheroids mirroring tissue-specific epithelial physiology. However, although these studies yielded SIX2+ NPCs cells with a higher efficiency than the work from Mae and colleagues, the necessity for agents as spinal cord precluded the generation of hPSCs-derived NPCs on a larger scale.

Other limitations at that point were highlighted by Morizane and collaborators who argued that the still not satisfying feasibility and efficiency to generate hPSCs-derived renal cells was caused by the fact that most of the initial procedures did not properly distinguish anterior from posterior IM at early steps of directed differentiation (Morizane et al., 2015). These findings and observations altogether laid the groundwork for the generation of the so-called kidney organoids. To date, various well characterized protocols to generate kidney organoids are available. The common theme used in these approaches is the activation of canonical Wnt signalling *via* CHIR in undifferentiated hPSCs to promote the formation of PS and IM derivatives from which the kidney derives (Takasato et al., 2014; Morizane et al., 2015; Garreta et al., 2019).

Morizane and colleagues reconsidered the application of BMP4, which was used in previous renal differentiation protocols to induce the anterior-posterior patterning of the PS. In this study, the authors hypothesized that posterior IM cells arise from the late-stage PS rather than from posterior PS, and highlighted the importance of recapitulating the timing of migration of mesodermal precursors out of the PS, a process that defines mesodermal anterior-posterior patterning during development. Following this rationale, they tested several doses and duration of CHIR treatments in order to effectively differentiate hPSC into late-PS cells to further increase the efficiency of IM formation (Morizane et al., 2015). This led to the now general notion that for optimal differentiation the duration of Wnt signalling determines whether anterior IM-derived UB or posterior IM-derived MM becomes the most dominant population as anteriorization of PS favors UB formation (Taguchi et al., 2014). This hypothesis was confirmed by Takasato and colleagues. In their study a shorter CHIR treatment generated predominantly anterior IM whereas longer exposure induced posterior IM (Takasato et al., 2015). Takasto and colleagues generated kidney organoids containing individual nephrons (∼100 nephrons/organoid) that further segmented into distal and proximal tubules, early loops of Henle and glomeruli containing podocytes elaborating foot processes. In this work the authors performed bulk RNA sequencing (RNA seq) for transcriptomically profile the extent of kidney organoid differentiation also comparing the matureness of the generated organoids with human embryonic kidneys and demonstrated that upon 25 days of differentiation kidney organoids transcriptomically resembled the first trimester gestational kidney (Takasato et al., 2015).

**FIGURE 2:**
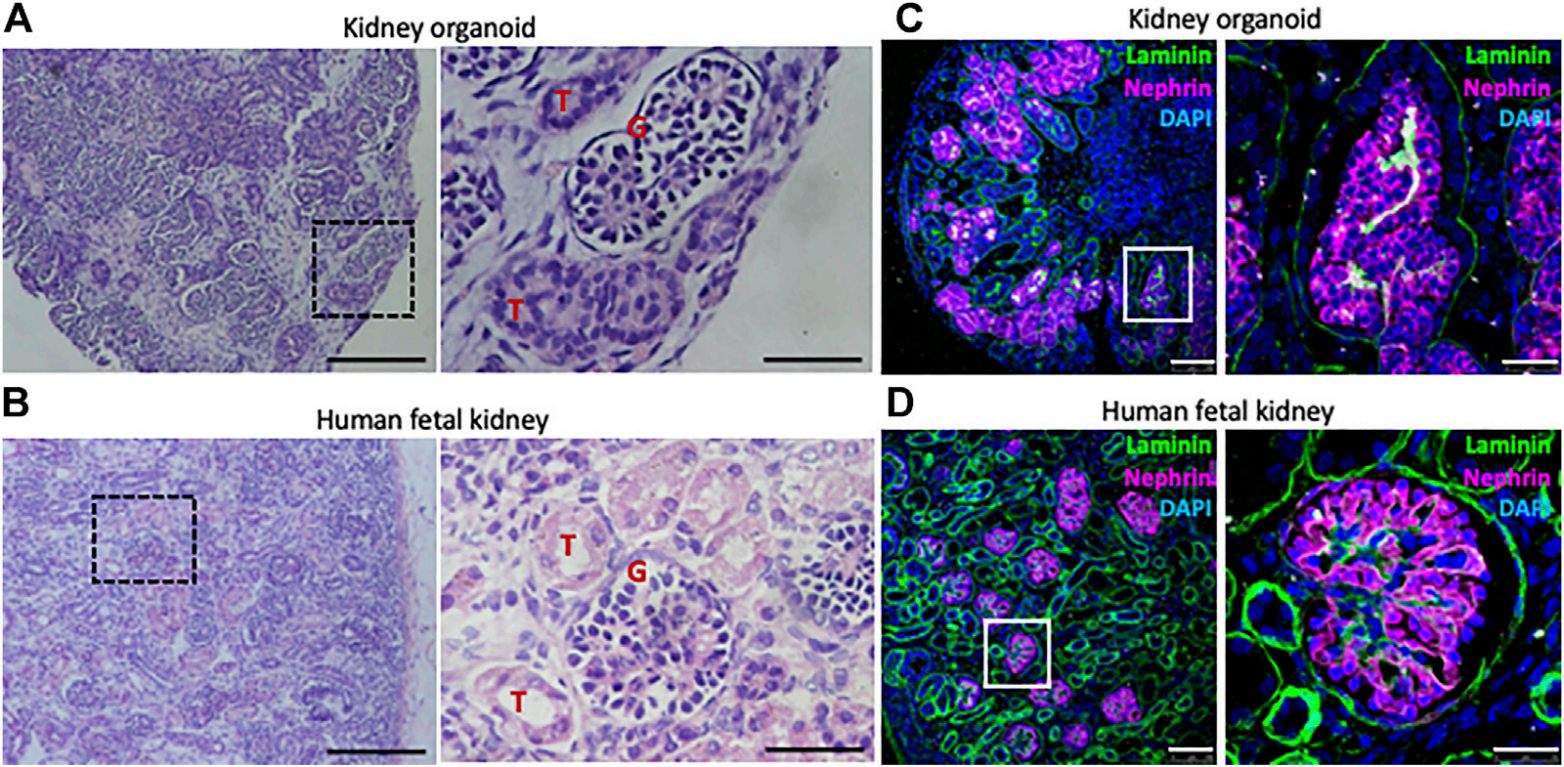
hPSC-derived kidney organoids display renal features that partially resemble those in human fetal kidneys. Histological analysis of kidney organoid **(A)** and 22 weeks of gestation human fetal kidney **(B)**. Higher magnifications of the boxed areas show details of glomeruli (G) and tubule (T) structures. Scale bars, 200 and 50 µm (higher magnifications). Immunohistochemistry of kidney organoid **(C)** and 22 weeks of gestation human fetal kidney **(D)** for the detection of the basement membrane protein Laminin and the podocyte marker Nephrin. Cell nuclei are counterstained with DAPI. Higher magnifications of the boxed areas show details of glomerulus found in kidney organoid compared to human fetal kidney. Interestingly, nephrin^+^ podocyte-like cells in kidney organoids are aligned on the basement membrane (laminin^+^) following a similar distribution as in the human fetal kidney. Scale bars, 100 and 25 µm (higher magnifications).

Morizane and colleagues reported that 3D culture conditions proved to be advantageous in order to generate tubular structures at a higher efficiency (Morizane et al., 2015). Furthermore organoid podocytes are being shown to express a range of proteins required for glomerular function (e.g., NEPHRIN, PODOCIN, PODOCALYXIN, SYNAPTOPODIN) which are nearly absent in conventional 2D podocyte cell lines (Yousef Yengej et al., 2020). In our own study we also observed that forcing cell-to-cell and cell-to-extracellular matrix interactions through the aggregation of NPC-derived hPSCs early during the time course of organoid formation results in the development of kidney organoids which upon 16 days in culture transcriptomically matched the second trimester human gestational kidney (Garreta et al., 2019) (Figure 2). Characterization of organoids via single cell RNA-sequencing (scRNAseq) confirmed the presence of developing podocytes, parietal epithelial cells, proximal tubules, loops of Henle, distal tubules, collecting ducts, and interstitial and endothelial cells. Kidney organoids reveal a great deal (20%) of off-target cells, mainly neural. Mostly, the common renal differentiation protocols generate kidneys matching second or trimester fetal kidneys (Combes et al., 2019; Subramanian et al., 2019).

Stromal populations have been identified in kidney organoids by immunofluorescence as well. The expression of *FOXD1 and MEIS1* indicated the presence of interstitial cells in kidney organoids (Takasato et al., 2014). Although not much attention is paid to stromal cells in the MM-derived organoids, Taguchi and colleagues identified them to be crucial for enhancing branching morphogenesis of the ureteric tree when incorporating mouse embryonic interstitium together with induced UBs and progenitors (Taguchi et al., 2014). Again, scRNAseq analysis highlighted underrepresented or missing cell types as mesangial cells, immune cells, glomerular endothelium, principal and intercalated cells (Combes et al., 2019; Yousef Yengej et al., 2020).

### 3.2 Major drawbacks in the field of kidney organoid derivation

Besides all the advances mentioned above, the kidney organoid field is already facing important drawbacks as organoids lack a nervous or immune system, a progenitor niche, and proper vascularization. Also, the assembly of UB and epithelial progenitors to generate a more developed model still needs to be achieved. As described earlier reciprocal interactions between the UB and the surrounding SIX2+ CM, however are essential to nephrogenesis. In this regard, the majority of procedures to derive kidney organoids from hPSCs first differentiate to kidney progenitors through the formation of NPCs that are then induced to epithelialize using a pulse of GSK3 inhibitor CHIR. Importantly, this single epithelialization induction differs from the process of differentiation in the native developing kidney, where cells at numerous stages of differentiation co-exist within the organ as recently described by the MacMahon laboratory (Lindström et al., 2018a). Following this logic, the laboratory of Oxburgh has recently shown that the asynchronous mixing of hPSCs-derived NPCs with epithelializing nephrons over time results in the generation of heterochronic organoids in where the proximal and distal nephron components preferentially derive from different cell populations. Furthermore, the resulting organoids were well vascularized when engrafted under the kidney capsule (Kumar Gupta et al., 2020).

Addressing the lack of vascularization is essential for several reasons. One is that renal diseases in the adult are mainly caused or aggravated by the immune system and widely based on damages of the vascular system (Tecklenborg et al., 2018). Kidney organoids lack an immune system and only contain an endothelial cell population instead of a fully developed vascular system, which might also be the reason why maturation cannot be pushed further and only leads to fibrosis. The improvement of vascularization of kidney organoids is studied more than the immune system, probably due to the notion that a vascular system is the key to more mature organoids by triggering cell communication. It is hypothesized that the lack of oxygen in the inner parts of the organoid are the main reason to why maturation stops at some point (Wörsdörfer and Ergün, 2021). We addressed the vascularization problem in our study by implanting organoids into chick immune-deficient chorio-allantoic membrane (CAM), a highly vascularized extraembryonic tissue previously used to grow tumoral cells or engrafting biomaterials. In our study, we took advantage of this system to enhance the proper organization of endogenous kidney organoid endothelial cells also exploiting the CAM assay as in ovo bioreactor to grow kidney organoids on demand. Our approach after 3 days of organoid implantation resulted in the invasion of multiple blood vessels from the CAM throughout kidney organoids. The circulation of chick blood within kidney organoids was clearly observed after 5 days of implantation, Overall, compared with *in vitro* counterparts, CAM-implanted kidney organoids exhibited glomeruli with an enlarged Bowman’s space and tubule-like structures with enlarged lumens (Garreta et al., 2019).

An alternative approach to provide a vascular component to hPSCs-derived organoids is to transplant them under the kidney capsule of immunodeficient mice profiting from the high vascularization capacity of this tissue. Two studies showed a maturation in transplanted podocytes and vascularization of glomeruli by the host respectively (Sharmin et al., 2016; Van Den Berg et al., 2018). Also it seems that fluid flow is contributing to an improvement of vascularization as it stimulates endothelial cells to invade podocyte populations (Musah et al., 2017). A recent study has explored this approach through the design of a 3D printed chamber at the millimeter scale to culture hPSC-derived kidney organoids under constant fluid flow that sufficed for the expansion of endothelial progenitors within the organoids and a higher degree of vascularization compared to the static culture conditions (Homan et al., 2019). This opens future perspectives in the bioengineering field to use chips, scaffold and other devices to culture organoids on in order to mimic filtration and tissue interface and recapitulate renal cell injury and proteinuria (Begum, 2019).

Recent studies also focused on the generation of kidney organoids with enhanced endothelial cell compartment such as reported from the Xia laboratory. They generated vascularized organoids by modulating Wnt signalling allowing to further control the relative proportion of proximal versus distal nephron segments for the production of vascular endothelial growth factor A (VEGFA), the major factor responsible for the maintenance of the glomerular vasculature (Eremina et al., 2003; Sison et al., 2010). More importantly, in this work the authors made use of single-cell RNA sequencing (scRNAseq) to further identify a subset of NPCs as a potential source of renal vasculature which was further supported by the revelation of Six1+CD31^+^ (SALL1+CD31^+^) cells (Low et al., 2019). In this regard, recent work from MacMahon laboratory has shown on the utility of single-nucleus droplet-based sequencing of the human fetal kidney for the identification of nephron, interstitial, and vascular cell types that together generate the renal corpuscles identifying factors predicting precursors or mature podocytes which express *FBLN2, BMP4, or NTN4,* in conjunction with recruitment, differentiation, and modelling of vascular and mesangial cell types into a functional filter (Kim et al., 2019). *In vitro* studies using primary cells from fetal kidneys proved that these factors exhibit angiogenic or mesangial recruiting potential also exerting inductive properties consistent with a key organizing role for podocyte precursors in kidney development. It will be interesting to challenge developing hPSCs kidney organoid to boost these processes.

Another technical challenge, regardless of the approach used to induce vascularization, is to recapitulate embryonic branching morphogenesis in kidney organoids. As predicted from mice (Costantini and Kopan, 2010), recent analysis in the human fetal kidney would suggest that the collecting duct tips should be marked by *RET* expression (Lindström et al., 2018b; Menon et al., 2018; Hochane et al., 2019). Nonetheless, there is no study showing *WNT9B* expression in kidney organoids nor any evidence for the existence of ureteric epithelium. In this regard, the Melissa Little group referred to the GATA3+CDH1+ structures encountered in kidney organoids as collecting duct (Takasato et al., 2015), however, in that same work RNA seq analysis did not reveal *WNT9B* or *RET* expression. Recently, the same group has referred to those structures as connecting segment (Little and Combes, 2019), which refers to the region of the nephron which bridges to the collecting duct epithelium (Little et al., 2007). Nonetheless, the same group did not find that this presumptive connective segment derives from SIX2+ cells as happens in mouse (Georgas et al., 2011) suggesting that this epithelium may arise from an ureteric epithelium during the time course of organoid formation. Therefore, the work from the Nishinakamura laboratory has been the only one providing a comprehensive overview on the distinct origins and developmental processes of the ureteric bud (Taguchi and Nishinakamura, 2017) taking advantage of the Hoxb7-GFP transgenic mouse line to establish a kidney reconstruction assay by modifying previously reported methods (Grobstein, 1953; Auerbach and Grobstein, 1958; Ganeva et al., 2011). Through the reaggregation of E11.5 MMs, including NPs and SPs with a wolffian duct (WD) or UB from E9.5, E10.5, and E11.5 the authors were able to show that UB or WD from the E11.5 embryo would robustly branch in front of WD from E10.5 and E9.5. Importantly, the authors employed the best culture condition supporting WD acquired branching capacity to define the biochemical cocktail inducing the maturation of WD progenitors into UB-like cells. This information allowed for the derivation of novel culture procedures for the differentiation of mouse ESCs into induced UB, which effectively reconstructed the higher-order structure of the embryonic kidney by their assembly with NPCs and isolated renal embryonic stromal cells. Then the authors translated these findings to applying the different procedures using hiPSCs. All in all, the work of Nishinakamura showed that reassembled organoids developed the inherent architectures of the embryonic kidney, including the peripheral progenitor niche and internally differentiated nephrons that were interconnected by a ramified ureteric epithelium. Thus, exploiting the selective induction on hPSCs together with the reassembly of the different cell types will be a powerful approach to recapitulate organotypic architecture in hPSC-derived organoids and to further assess the role of human UB branching epithelium as an organizer of tissue geometry and cell viability during human embryonic kidney development. A summary of recent published reports on the generation of hPSC-derived kidney progenitors and kidney organoids is compiled in Table 1.

**TABLE 1:** Major approaches and protocols for directed differentiation of hPSCs into renal progenitor cells and kidney organoids.

Author and year	Stem cell line	Stepwise differentiation protocol			hPSC-derived renal cells by 2D or EBs differentiation	Generation or kidney organoids	Cell types within kidney organoids	Functional and validation assays
Song, B 2012	hiPSCs from human kidney mesangial cells	3days suspension culture of iPSCs colonies with DMEM-F12 + 2.5% FBS + AA + BMP7+RA			Podocyte progenitor cells expressing Podocin, Synaptodin, PAX2, WT1, NPHS1	N/A	N/A	Permeability assay showed endocytic uptake of FITC- albumin.
		7–8 days on gelatin coated monolayer with same suplementation						Integration within glomerular aggregates of embryonic mouse kidney explants.
Narayanan, K 2013	(HUES)-7 hESCs	20 days culture on Matrigel with REGM +0.5% FBS + BMP2+BMP7			Renal proximal tubular-like cells expressing AQP1 and a markers profile similar to HPTCs	N/A	N/A	Contribution to CK18-positive tubular epithelia when injected into mouse cortex kidney explants.
								Formation of tubular structures after implantation subcutaneously into immunodeficient mice.
								Response to PTH and GGT activity into biorreactor cultures for further bioartificial kidney applications.
Mae, S 2013	201B7 hiPSCs from dermal fibroblasts	Stage 1: 2days AA + CHIR	DMEM/F12 + 2%FBS DMEM/F12 + B27	EB method Colony method (Matrigel) Single-cell method (Collagen I) Serum-free single-cell method (Collagen I)	IM cells expressing OSR1 that further differentiated into polarized tubule-like structures expressing LTL/LAMININ/AQP1, few UB cells expressing DBA, glomerular cells expressing PODXL/PNA and gonadal or adrenocortical cells expressing GATA4/GATA6/HSD3β	N/A	N/A	Differentiation of IM cells into renal tubular like cells after their transplantation into the epididymal fat pads of immunodeficient mice
		Stage 2: 8days BMP7+CHIR	DMEM/F12 + 10%KSR DMEM/F12 + B27	EB method Colony method (Matrigel) Single-cell method (Collagen I) Serum-free single-cell method (Collagen I)				Integration to form tubular like structures when cocultured with mouse metanephric tissue.
Xia, Y 2013	H1 hESCs and 201B7 hiPSCS from dermal fibroblasts	Monolayer cell culture on matrigel and DMEM/F12 supplemented with: 2days BMP4+FGF2 and insulin → Mesoderm			UB progenitor-like cells expressing PAX2/LHX1 and a markers profile similar to UB	N/A	N/A	Integration into the UB tip and truck when re-associated with mouse embryonic kidneys
		2days RA + AA and BMP2 → IM and UB lineages						
Takasato, M 2014	HES3 hESCs, H9 hESC and CRL2429 C11 hiPSCs from foreskin fibroblasts	Matrigel culture and APEL basal media suplemented with: 2days AA BMP4 → PS 4days FGF9 → IM 11dayays FGF9+BMP7 and RA → MM and UE			Stepwise differentiation from PS cells expressing MIXL1/LHX1 to IM cells expressing OSR1/PAX2/LHX1 to finally obtain both: MM cells expressing SIX2/WT1/GDNF/HOXD11 and UE cells expressing C-RET/HOXB7	N/A	N/A	Reaggregation assays with dissociated mouse embryonic kidneys showed specific integration into only MM and UE compartments
					Later differentiation timepoints showed UE structures expressing ECAD surrounded by MM cells			
		Matrigel culture and APEL basal media suplemented with: 2days CHIR → PS 10days FGF9 → IM 6days No growth factors → MM and UE			Same stepwise differentiation but with a faster induction of kidney markers and a more prolonged expression of MM genes.	Enzymatically dissociation into single cells, pelleted and cultured on a filter membrane at an air–media interface with DMEM 10% FCS during 4days.	ECAD + tubules expressing UE markers PAX2/AQP2 and proximal tubule markers AQP1/SLC3A1.	Reaggregation assays with dissociated mouse embryonic kidneys showed integration into compartments of the developing kidney including UE, early nephron/renal vesicles and nephron progenitor mesenchyme
					Additional GATA3 expression in UE cells already expressing PAX2			
					MM cells expressing PAX2 and condensing tightly around the UE tips. Presence of renal stroma cells WT1- and HOXD11+.		MM cells expressing WT1/PAX2 and surrounding the ECAD + UE. MM also contained renal vesicles expressing JAG1/ECAD.	
					Later differentiation showed UE structures expressing ECAD and surrounded by MM. This MM contained nephron/renal vesicles like structures expressing CDH6/JAG1+.			
Lam, A. Q. 2014	H1, H9, CHB8-H2B-GFP hESCs and hiPSCs from human foreskin or human dermal fibroblast	Geltrex culture and RPMI basal media suplemented with: 2days CHIR → ME 4days FGF2+RA → IM 5days FGF9+AA or 7days No growth factors		Stepwise differentiation from mesendoderm cells expressing T/MIXL to IM cells expressing PAX2/LHX1 that:	After growth factors withdraval spontanously induced tubule-like structures expressing LTL/KSP/NCAD	N/A	N/A	Re-aggregation assays of tubular-like cells with dissociated mouse embryonic kidneys showed integration in both metanephric interstitium and murine laminin-bounded structures but not in murine tubular-like structures.
								*In vivo* implantation of tubular cells beneath the kidney capsule of immunodeficient mice generated human growths expressing AQP1.
				After FGF9 + AA treatment induced cap mesenchyme NPCs expressing SIX2/SALL1/WT1				CHIR treatment in SIX2+ cap mesenchyme nephron progenitors induced tubulogenesis similar to an *in vivo* scenario.
								Re-aggregation assays of SIX2+ cap mesenchyme NPCs showed organization into clusters of cells expressing LTL
Taguchi, A 2014	Based in protocol for mouse ESCs, hiPSCs from dermal fibroblasts were differentiated	Suspension culture and: 1day Serum free medium with BMP4+Y27632 → EBs 2days Serum free mediumwith AA + Fgf2 → Epiblast 6days BC10 medium with BMP4+CHIR → Posterior Nascent M 2days ABC3R medium with AA + BMP4+CHIR + RA→ PIM 3days ABC3R media with CHIR + FGF9 → MM			Majority of IM cells expressing WT1 at day 11.	Reagregation on day 14 with mouse embryonic spinal cords and cultured on a air-fluid interface of a polycarbonate filter	Early glomeruli expressing WT1/NPHS1.	Chimeric orgnaoid was used as a functional read out
							Proximal tubules expressing CDH6.	
					MM cells expressing WT1/PAX2/SALL1 at day 14.		Pistal tubules expressing CDH1 structures.	
							Some MM cells expressing SALL1/PAX2.	
Imberti, B 2015	SC101 A1 hiPSCs from foreskin fibroblasts and in house derived hiPSCs from human fibroblast	DMEM/F12 supplemented with: 1day RA + PI3K inhibitor + RhoA inhibitor 2days RA + PI3K inhibitor + RhoA inhibitor + AA → ME 3days RA + PI3K inhibitor + RhoA inhibitor → IM 13days FGF2+BMP7+GDNF → MM differentiating into NPCs			MM cells expressing WT1/PAX8/PAX2/SIX2/SALL1 that progressively adquired renal progenitor phenotype specific markers such as NCAM, CD133, CD24 or AQP1 and formed nephrogenic like peripheral patterns.	N/A	N/A	Day 12-differentiated cells were intravenously infused into cisplatin-induced AKI mouse model. Four days after infusion, human cells were found integrated into proximal murine tubuli and a significant improvement of renal function in terms of reduced BUN levels and improved histological observations such as reduction of cell swelling, less cast deposition, integrity of brush borders and reduced cell necrosis
Morizane, R 2015	H9 hESCs, hiPSCs from dermal fibroblasts	Culture on Geltrex and Advanced RPMI 1640 suplemented with: 4days: 8 CHIR for ESCs; CHIR + noggin for iPSCs. → LPS 3days: AA → PIM 2days: FGF9. Next steps on 2D or in 3D kidney organoids → MM 2days: CHIR + FGF9 → Pretubular aggregate 3days: FGF9 → RV 7–14days: no growth factors → Nephrogenesis			NPCs expressing SIX2/SALL1/WT1/PAX2 on day 9 of differentiation that further differentiated on day 11 in RVs expressing PAX8/LHX1/HFN1b/BRN1. RVs finally developed into elongated epithelial nephron structures including glomerular podocytes expressing NPHS1/PODXL, proximal tubules expressing LTL/CDH2 and loops of Henle/distal tubules expressing ECAD/CDH1/UMOD/BRN1	Alternatively to 2D differentiation on day 9, NPCs were replated in ultra-low attachment round bottom plates, pelleted and mantained in 3D suspension culture. Spherical aggregates underwent nephrogenesis.	Clusters of podocyte-like cells expressing WT1/PODXL/NPHS1 connected to tubular structures with proximal tubules expressing LTL/CDH2/AQP1, loops of Henle expressing CDH1/UMOD and distal tubules expressing only CDH1.	Supression of proximal tubules by Notch signalling inhibitor DAPT as previous developmental studies in mice.
								Tubular toxicity response to cisplatin and gentamicin.
Freedman, BS (2015)	H9 and WA09 hESCs, hiPSCs derived from foreskin and dermal fibroblasts.	Sandwiched culture between matrigel 3days: mTeSR1 medium → Cavitated spheroids 1.5days: RPMI suplemented with CHIR → ME 11.5days: RPMI suplemented with B27 → from MM to RVs Onwards no growth factors → Mature tubular organoids			Full protocol carried on 3D culture: from cavitated spheroids to kidney organoids.	hPSCs were dissociated and sandwiched between two layers of dilute Matrigel. Embebed colonies were induced to form cavitated spheroids that further differentiated into kidney organoids.	Sequential presence of IM cells expressing PAX2 on d7, MM expressing SIX2/WT1 on d14 and renal vesicles expressing PAX2/LHX1 on d22 that final differentiated into renal cell types including podocyte-like cells expressing WT1/PODXL/SYNPO, proximal tubular cells expressing CDH1/LTL, distal tubular cells tubules expressing only CDH1 and endothelial cells expressing CD31/vWF+. Off-target neuron-like cells expressing TUJ1 were also observed. Tubules expressed nephron progenitor/renal vesicle markers including LHX1 and PAX2. MM cells expressing SIX2 appear adjacent to organoids.	Selective uptake of dextran cargoes in tubules.
								Tubular toxicity response to cisplatin and gentamicin. Disease modelling when PKD1, PKD2 and PODXL are depleted by CRISPR.
								Implantation of organoid cells into the cortex of immunodeficient mice showed 3 weeks survival and expression of LTL at similar intensities as neighboring mouse tubules.
Takasato, M 2015	CRL1502-C32 hiPSCs derived from fibroblasts	Culture on matrigel and APEL basal medium suplemented with: 4days: CHIR → Anterior and posterior IM 3days: FGF9+heparin → IM 11–18days: CHIR for 1 h, then 5days FGF9+heparin and finally no growth factors → Nephrogenesis into self-organizing organoid.			Posterior IM cells expressing GATA3/HOXD11 on day 7. Alternatively to organoid generation cells were further differentiated in 2D to UE expressing GATA3/PAX2/ECAD or MM expressing only PAX2 and its derivatives expressing PAX2/ECAD. Preferential induction of UE vs. MM was tested with different extents of CHIR treatment in combination with different growth factors.	On day 7 cells were dissotiated, pelleted and transferred to a pore polyester membrane of a transwell culture system.	Early podocytes expressing WT1/NPHS1. Proximal tubules expressing CDH1/LTL. Early loops of Henle expressing CDH1/UMOD. Distal tubules expressing only CDH1 that were associated with a CD network expressing PAX2/GATA3/ECAD. Cortical stroma cells expressing FOXD1/MEIS1. Medullary stroma cells only expressing FOXD1. Endothelial cells expressing CD31/KDR/SOX17.	Selective uptake of dextran cargoes in tubules.
								Tubular toxicity response to cisplatin.
								Comparative RNA-Seq analysis clustered kidney organoids at d11 and d18 with first trimester human fetal kidney.
								TEM showed the presence podocyte-like cells aligned on a basement membrane and developing primary and secondary cell processes.
Taguchi, A 2017	201B7 hiPSCs from dermal fibroblast	NPCs induction from hiPSCs adapted from Taguchi, et al., 2014 1day Aggregation in V-bottom plates Y27632 + AA + bFGF → EBs 6days in U-bottom plates CHIR+Y27632 → M 2days ABC3R medium with AA + BMP4+CHIR + RA+Y27632 → PIM 3days CIF medium with CHIR + FGF9+Y27632 → MM			NPCs defined by positive expression of ITGA8 and negative for PDGFRA expression.	N/A	N/A	Reconstitution assays of induced NPCs and UB cells from mESCs with primary stromal progenitors generated murine organoids with nephrons interconnected by branched epithelium. These assays are NOT characterized with human cells in this report.
		UB induction and WD maturation from hiPSCs in basal media DMEM/F12/B27 supplemented with: 1day Y27632 + AA + BMP4 in V-bottom plates → EBs 1.5days CHIR+BMP4 in U-bottom plates → M 2days RA + FGF9+LDN193189 + SB431542 → Anterior IM 2days RA + CHIR + FGF9+LDN193189 → WD 2days Y27632 + RA + CHIR + FGF9+FGF1+LDN193189 + Matrigel → 3D WD maturation 2days previous components + GDNF → 3D WD maturation 2days previous components without FGF9 → UB organoid			WD precursors expressing CXCR4 and KIT markers	Sorting of CXCR4+/KIT+ and aggregation in V-bottom plates at day 6.5 of differentiation. For branching, UB organoids on day 12.5 were embedded in transwell inserts and cultured in DMEM/F12 suplemented with Matrigel+10%FBS + RA + humanRspondin1+GDNF + FGF1+FGF7+LDN193189	UB cells expressing HNF1b, E-cadherin, and CALB1 that generated a branched organoid. Ureteric epithelium showed tips with cells expressing SOX9 and stalk regions with cells expressing CK8. Detailed analysis of the tip region identified the typical ampulla or dichotomous bifurcation with cells expressing PAX2/ECAD.	Evaluation of PAX2 knockout hiPSCs for NPCs or UB induction elucidated the role of this transcription factor into MET of WD precursors.
Garreta, E 2019	ES[4], H1, H9 hESCs and CBiPSsv-4F-40 hiPSCs derived from CD133+ cord blood cells	Initiation in VTN coated plates. Advanced RPMI 1640 basal medium was suplemented with: 4days CHIR → PPS 1day AA + FGF9 → IM Generation of IM commited spheroid 3days FGF9+CHIR →→ NPCs 5days FGF9 → RV 8days No growth factors → Nephrogenesis			PPS cells expressing T that further after CHIR treatment that further differentiated into IM commited cells expressing PAX2 and other markers such as OSR1/HOXD11/GATA3	IM committed cells were dissociated and agregated into low attachment round bottom plates for organotypic culture	Just after final FGF9 treatment organoids presented patterned RVs with cells expressing PAX2/WT1/LHX1/PAX8/HNF1β/ECAD/BRN1.	TEM showed the presence podocyte-like cells with deposition of a basement membrane and developing primary and secondary cell processes. Presence of brush borders and high mitochondrial content was observed in epithelial tubular-like cells.
								Comparative RNA-Seq analysis clustered kidney organoids with second trimester human fetal kidney.
							RVs finally developed at the end of differentiation into nephron-like structures that were segmented into proximal tubules expressing LTL/AQP1/SLC3A1 loops of Henle expressing ECAD/UMOD distal tubules only expressing ECAD+ glomeruli expressing PODXL/PODOCIN/NPHS1/WT1	Reagregation assays with mouse embryonic kidney cells showed how differentiated cells after CHIR + FGF9 treatment integrated into mouse nascent nephron structures.
								Recapitulate complex nephron patterning events such as distalization after b-catenin induction or severe loss of glomeruli and proximal tubuli after Notch signalling disruption.
								Enhanced tubular differentiation under organoid culture favouring oxidative phosphorylation.
								Implantation of organoids into CAM promoted vascularization which enhanced maturation of nephron structures and was able to deliver injected chemicals such as cisplatin.
								Organoid differentiation was enhanced when IM commited cells were differentiated in softer substrates similar to the stiffness of *in vivo* microenviroments.
Low, J. H. 2019	H9 and H1 hESCs and GM10287 hiPSCs derived from fibroblasts	Culture on Matrigel and Advanced RPMI 1640 medium suplemented with: 4days CHIR → PS 3days without growth factors → IM 2days FGF9+CHIR → NPCs 1day FGF9+Y27632 → Nephrogenic organoids 9days FGF9 → Vascularized Kidney organoids			PS cells expressing T/MIXL1 that differentiated to IM cells expressing HOXD11/WT1 which finally generated NPCs expressing SIX2/SALL1	NPCs on day 10–12 were dissociated, aggregatedand pelleted in U-bottom plates.	Transient pretubular aggregates expressing SIX2/SALL1/LHX1/PAX8 that further developed into nephron containing: podocytes expressing NPHS1/PODXL/VEGFA, proximal tubular cells expressing LTL, medial tubular cells expressing JAG1 and distal tubular cells expressing CDH1.	Coculture with HUVECs showed integration of exogenous endothelial cells with resident vascular network increasing organoid vascularization.
								VEGFR inhibitors compris∫ed organoid vascular networks
							Vascular progenitors expressing KDR that further adquired CD31/CD34 expression and placed alongside nephron structures.	scRNA-seq suggested a subset of NPCs with vascular progenitor-like property as the origin of organoid vasculature.
								CHIR treatments altered neprhon patterning by promoting tubulogenesis.
								*In vitro* dextran uptake by proximal tubules.
								Implantation beneath the renal capsule of immunocompromised mice improved organoid differentiation and vascularization. *In vivo* filtration and reabsorption by implanted organoids was shown after perfusion of dextran into murine vasculature.
								Cystogenesis recapitulation by kidney organoids differentiated from ARPKD hiPSCs
Yoshimura, Y 2019	201B7 hiPSCs derived from fibroblasts and RN7 derived from blood cels	Starting from NPCs differentiated from hiPSCs as Taguchi, A 2017 protocol. NPCs ITGA8+/PDGFRa- population was sorted and cultured in U-bottom plates with serum free medium suplemented with: 1day CHIR + Y27632 → Pretubular aggregate aggregated cells were transferred to a transwell insert 2days FGF9+IWR-1+SB431542 + RA → Proximalized RV 6-9days IWR-1+SB → Podocytes			NPCs first differentiated in pretubular aggregates expressing LHX1 that further acquired CDH1 expression generating RVs. RVs differentiated up to 90% of podocytes expressing NPHPS1.	N/A	N/A	RNA-seq showed similar gene expression profile to human adult podocytes.
								Induced podocytes also showed higher protein expression levels of podocyte-related proteins than inmortalized podocytes or podocytes derived from convectional kidney organoids.
								PAN treatment induced podocytes injury as *in vivo*.
								TEM showed protrusions of the basolateral domain expressing NPHS1 on their surface membrane. Slit diaphragm–like structures were also detected.
Kumar Gupta, A 2020	H9 hESCs and WTC11 hiPCs derived from fibroblasts	Starting from NPCs differentiated from hiPSCs as Morizane et al., 2015 protocol. Organoids were generated and cultured in air-liquid interface with APEL2 basal medium suplemented with: 4days BMP7+FGF9+Heparin 7days No growth factors			N/A	NPCs derived by Morizane protocol were dissociated, aggregated in a small volume which was finally dropleted on polycarbonate filter to generate a air-liquid interface culture system. After two days, differentiating organoids were mixed with newly differentiated NPCs and reaggregated to finally generate heterochronic organoids. Control organoids were not mixed with new NPCs	Proximal tubuli expressing LTL distal tubuli expressing BRN1/CDH1 collecting duct expressing CDH1/GATA3/DBA Podocytes expressing PODXL/WT1 endothelial network expressing CD31 Pericytes expressing PDGFRβ Remarkably heterocronic organoids displayed the double number of structures stained for each molecular marker and showed less remnant undifferentiated NPCs expressing SIX2 than control organoids.	Engrafted heterocronic organoids under the kidney capsules of immunocompromised mice showed enhanced maturation and functional vascularization.
								Systemic perfused FITC-IB4 labeled endotelial vascular cells in close contact with organoid podocytes.
								Dextran was accumulated in organoid tubules after after its systemic perfusion.

Comparison of recent protocols that have been used to generate renal cells and eventually kidney organoids from hPSCs, including the following information: Schematic description of protocol steps in terms of duration, growth factors and lineages obtained, Renal cells induced by each protocol prior organoids formation in two-dimensional or Embryonic Body differentiation prior to organoids generation; Description of the method used to generate kidney organoids *in vitro*; Type of renal cells and structures appearing into organoids and finally; Assays performed to either improve the characterization or validate functionally the renal derivates obtained after each differentiation protocol. AA, activin A; AKI, acute kidney injury; AQP1, aquaporin 1; AQP2, aquaporin 1; ARPKD, autosomal recesive policystic kidney disease; bFGF, basic fibroblast growth factor; BMP, bone morphogenetic protein; BRN1, POU, Class 3 Homeobox 3; BUN, blood urea nitrogen; CD, collecting duct; CD133, cluster of differentiation 133; CD24, cluster of differentiation 24; CD31, cluster of differentiation 31; CD34, cluster of differentiation 34; CDH1, cadherin 1; CDH2, cadherin 2; CDH6, cadherin 6; CHIR, CHIR99021 inhibitor; CK18, cytokeratin 18; CXCR4, C-X-C chemokine receptor type 4; d, days; DBA, lectin Dolichous biflorus agglutinin; EB, embryoid body; ECAD, E-Cadherin; FBS, fetal bovine serum; FGF, fibroblast growth factor; FGF1, fibroblast growth factor 1; FGF2, fibroblast growth factor 2; FITC-IB4, fluorescein labeled Griffonia Simplificata Isolectin B4; FITC, fluorescein; FOXD1, forkhead box 1; GATA3, GATA, binding protein 3; GDNF, glial cell derived neurotrophic factor; GGT, γ-glutamyl transferase; hESCs, human embryonic stem cells; hESCs, human pluripotent stem cells; hiPSCs, human induced pluripotent stem cells; HNF1β, hepatocyte nuclear factor-1, beta; HOXD11, homeobox D11; HPTCs, human primary proximal tubular cells; HSD3β, 3β-hydroxysteroid dehydrogenase; IM, intermediate mesoderm; ITGA8, integrin subunit alpha 8; IWR-1, tankyrase inhibitor; JAG1, jagged 1; KDR, kinase insert domain receptor; KSP, KSP-cadherin; KSR, knockout serum replacement; LDN193189, BMP, signalling inhibitor; LHX1, LIM, homeobox 1; LPS, late primitive streak; LTL, lotus tetragonolobus lectin; ME, mesoderm; MEIS1, meis homeobox 1; MET, mesenchymal–epithelial transition; MIXL1, Mix paired-like homeobox; MM, metanephric mesenchyme; N/A, not available; NCAD; N-Cadherin; NCAM, neural cell adhesion molecule; NPCs, nephron progenitor cells; OSR1, odd-skipped related transcription factor 1; PAN, puromycin aminonucleoside; PAX2, paired box 2; PAX8, paired box 8; PDGFRa2, Platelet Derived Growth Factor Receptor Alpha; PDGFRβ, Platelet-derived growth factor receptor beta; PIM, posterior primitive streak; PNA, peanut agglutinin; PODXL, podocalyxin; PS, primitive streak; PTH, parathyroid hormone; RA, retinoic acid; RhoA, ras homolog family member A; RV, renal vesicle; SALL1, spalt-like transcription factor 1; SB431542, TGFβ RI, Kinase inhibitor VI; scRNA-seq, Single cell RNA, sequencing; SIX1, SIX, homeobox 1; SIX2, SIX, homeobox 2; SLC3A1, solute carrier family 3 member 1; SOX9, SRY-Box transcription Factor 9; T, brachyury transcription factor; TEM, transmission electron microscopy; TUJ1, neuron-specific class III, beta-tubulin; UE, ureteric epithelia; UMOD, uromodulin; VEGFA, vascular endothelial growth factor A; VEGFR, vascular endothelial growth factor receptor; vWF, von willebrand factor; WD, wolffian duct precursors; WT1, Wilms tumor 1 gene; Y27632, ROCK, inhibitor.

### 3.3 Genome editing in hPSCs derived kidney organoids

#### 3.3.1 Using CRISPR/Cas9 to generate reporter cell lines to understand kidney development

Genetic lineage tracing using transgenic mice with fluorescent reporter genes have significantly contributed to our knowledge of mammalian kidney development and physiology (Humphreys and DiRocco, 2014). In those studies, fluorescence labelling allowed for the identification and real time monitoring of specific renal cell types during development or disease progression *in vivo* (Boyle et al., 2008; Zhang et al., 2020). Although this approach is not affordable in the human context it is possible to generate kidney organoids from hPSCs reporter lines to address similar questions so far addressed in the mice system. To date, accumulative findings on the generation of kidney organoids highlight the utility of this cell cultures as *in vitro* models to study mammalian kidney development, multicellular organization and physiological function. In this context, the possibility to perform functional genetic studies help to elucidate molecular mechanisms underlying organ development and disease. Nowadays, the use of targeted nucleases for genome editing facilitates our capacity to manipulate the genome of hPSCs and exploit these cellular platforms to virtually any desired cell type, including organoids. Among current genome editing technologies, this review focus on the utility of the CRISPR/Cas9 system in kidney organoids as an amenable system on which kidney differentiation and maturation can be monitored in living cells using fluorescently tagged kidney lineage markers. Indeed, in the near future our reliance on these cellular models may not only be restricted in their utility in differentiation studies but also help us to study the impact of renal cell de-differentiation under disease contexts (i.e., podocyte de-differentiation is a hallmark of CKD, as well as the de-differentiation of tubular epithelial cells upon acute injury, among others).

In the recent years the work of Boreström and colleagues exploited CRISPR/Cas9 to generate three kidney-specific reporter cell lines (Boreström et al., 2018). These include the derivation of two single reporters (SIX2- GFP and NPHS1-GFP), and one dual reporter cell line (SIX2-GFP/NPHS1-mKate). Making use of SIX2-GFP and NPHS1-GFP reporter cell lines the authors were able to monitor the emergence and maturation of kidney progenitor cells and podocytes, respectively. The percentage of fluorescent positive cells was used as a quantitative readout to redefine existing procedures for kidney organoid derivation. Furthermore, the authors made use of the NPHS1-GFP reporter cell line as a surrogate of podocyte health by assessing the impact of several insults on podocyte de-differentiation (measuring GFP loss). On balance, this panel of hPSCs reporter lines allowed the authors to establish an approach to monitor kidney differentiation, glomerular maturation, and podocyte performance in living cells.

In a more extensive approach Howden and colleagues targeted the *SIX2 locus* of hiPSCs to generate both reporter and lineage tracer cell lines (Howden et al., 2019). In line with similar lineage tracing studies in mice, the panel of CRISPR engineered hiPSCs was used to perform fate-mapping studies during kidney organoid differentiation. Importantly, the authors took advantage of these cell lines to underscore if the *SIX2* progeny contributes to nephrogenesis in developing hPSCs-kidney organoids. To achieve this CRISPR/Cas9 was used to generate reporter lines by inserting an EGFP expression cassette at the *SIX2 locus* of hiPSCs which upon characterization were differentiated into kidney organoids. By monitoring EGFP fluorescence expression during differentiation the authors were able to dissect the emergence of the EGFP+/SIX2+ cell population soon after kidney organoid formation and to demonstrate the presence of SIX2+ cells until later stages of differentiation. Then, CRISPR/Cas9 was used to generate a lineage tracer line by inserting both a *Cre* recombinase gene and a dual-fluorescence expression cassette that included a loxP-flanked EGFP gene adjacent to a mCherry gene. These constructs were inserted into the *SIX2* and *GAPDH loci* of hiPSCs, respectively. Monitoring mCherry and EGFP fluorescence during kidney organoid differentiation allowed to determine how the mCherry+/SIX2+ cell population gives rise to nephron epithelial cell types but not to presumptive ureteric epithelium (EGFP+) as previously reported in mice (Georgas et al., 2011). Interestingly, this study showed that some mCherry+/SIX2+ cells can give rise to a subset of the renal stroma, which points out a possible species divergence compared to mice. Finally, the authors made use of a CRISPR/Cas9 knock-in approach to generate an inducible lineage tracer line by inserting a tamoxifen-inducible version of the *Cre r*ecombinase (CreERT2). By controlling the labelling of SIX2+ cells at different time points during organoid differentiation the authors ascertain whether the SIX2 lineage contributes to nephrogenesis. This approach allowed the authors to induce *SIX2* expression at later time-points during differentiation, further showing the absence of mCherry + cells into nephron developed structures but their detection in the surrounding interstitium. Then the authors made use of CHIR at later stages of kidney organoid differentiation and treated cells with tamoxifen further showing the presence of mCherry + cells into nephron structures. Based on these results the authors concluded, that in contrast to kidney development *in vivo*, kidney organoids lack a self-renewal progenitor niche with active nephron-forming capacity.

Later on, the same group reported the application of CRISPR/Cas9 to develop a wide panel of reporter lines in a more throughput manner (Vanslambrouck et al., 2019). Towards this aim the authors used a single step protocol of simultaneous somatic reprogramming using episomal vectors together with CRISPR/Cas9 mediated genome editing for the generation of gene edited hiPSCs (Howden et al., 2018). Interestingly, the authors utilized a variant of Cas9 in order to minimize unwanted mutations derived from NHEJ DNA repair. This variant consisted of a Cas9 fused to a peptide derived from the human Geminin protein which mediates degradation of the chimeric protein (Cas9-Gem) during the G1 phase of the cell cycle at which cell stage NHEJ predominates over DSB. Thus, the absence of Cas9-Gem prevents from undesired mutations. Using this elegant approach, the authors expanded their previous reported panel of reporter cell lines up to ten different hiPSCs reporter lines that were further differentiated into kidney organoids. In this same study the authors further explored *SIX2* positive presumptive NPCs to ascertain on the role of this transient cell population in kidney organoids. In order to further characterize, visualize, and isolate this SIX2+ cells, additional hiPSC reporter lines were generated by targeting the nephron progenitor marker *CITED1*. This way the authors generated new single CITED1mCherry and double SIX2EGFP:CITED1mCherry hiPSC reporter lines. Remarkably, CITED1+/mCherry + cells were detected in higher amounts upon posterior primitive streak induction in agreement with the observed *CITED1* expression pattern in developing mice (Boyle et al., 2007). In the same work the authors also developed single reporter hiPSCs to further explore on the contribution of differentiated organoid cell types *in vivo*, including proximal tubular cells and podocytes, further showing correct engraftment of reporter cells into corresponding nephron compartments upon kidney organoid transplantation.

Another challenge in kidney organoid differentiation is the maintenance of a ureteric tip environment for NPCs survival and self-renewal. *In vivo* UB branches into a contiguous ureteric epithelium to which distal nephrons are connected. To allow further characterization of this ureteric and late distal epithelium within kidney organoids, in this same work the authors generated both a single GATA3mCherry hiPSC reporter line and double GATA3mCherry/MAFBmTagBFP2 reporter lines. Consistent with previous data, during differentiation GATA3+mCherry + cells were found into interstitial and nonepithelial organoids compartments that were frequently associated with NPHS1+ podocytes of the glomeruli. Furthermore, live imaging during kidney differentiation of the double reporter line evidenced coincidence of both GATA3+Cherry+ and MAFB + BFP2+ populations within a given organoid and dichotomy in onset of its labeled gene expression. These data provide novel insights into the debate around whether both human iPSC-derived NPCs and UB progenitors can be simultaneously generated in a single differentiation despite their divergent lineage origin.

On balance, the use of reporter hPSCs for the generation of kidney organoids allows to dissect and monitor human kidney morphogenesis in real time. The convergence of hPSCs reporter cell lines together with kidney organoid technology is expected to improve existing differentiation protocols with respect to cell type maturation and minimization of off-target populations. In this regard recent findings from the Greka laboratory (Subramanian et al., 2019) recently show on the impact of kidney organoid transplantation as a new approach to diminish the presence of off-target cells using scRNAseq. It will be interesting to further exploit these same approaches making use of hPSCs reporter cell lines and using strategies for further hPSCs lineage tracing to further ascertain the transcriptomic profile of the derived cells. The convergence of these methods may help to categorize organoids protocols with regards to the production of specific cell types and to predict fidelity and reproducibility.

#### 3.3.2 CRISPR/Cas9 application in kidney organoids: Modelling renal disease

Another application of CRISPR gene editing in kidney organoids is to interrogate and dissect human lineage relationships *in vitro* during kidney disease. This approach allows to perform functional experiments to either validate already known kidney disease related genes but also to identify new genes and cellular pathways malfunctioning during kidney disease. Specifically, by using CRISPR one can introduce mutations at candidate genes and, consequently, generate mutated hPSCs to be compared versus untargeted hPSCs controls that will share the same genetic backgrounds. In this manner, by differentiating mutant and wild type counterparts towards kidney organoids is not possible to study phenotypic and molecular differences arising from the genetic disorder.

Following this rationale Freedman and colleagues pioneered the application of CRISPR to introduce loss-of-function mutations into polycystin 1 (PKD1), polycystin 2 (PKD2) and podocalyxin (PODXL) genes. *PKD1* and *PKD2* genes were targeted to model polycystic kidney disease (PKD), which cause autosomal polycystic kidney disease, a condition which leads to end-stage renal failure due to expansion of fluid-filled cysts in the kidney. The same group targeted also the *PODXL* gene to investigate its possible implication into diseases which are based on a massive loss of protein due to defects in the kidney basal membrane as focal segmental glomerulosclerosis or congenital nephrotic syndrome (Freedman et al., 2015). In this initial study, although only a low percentage of *PKD* mutant organoids formed cysts and the disease mechanism still needs to be clarified, they proved that a functional model can be achieved this way. The authors furthermore generated a CRISPR/Cas9 engineered iPS PODXL knock-out line, which revealed that *PODXL* defective organoids exhibited an impairment of junction organization between podocytes-like cells.

Interestingly, a follow up study by the same group made use of kidney organoids derived from *PKD1* and *PKD2* mutant hPSCs to identify modulators of early PKD cystogenesis (Cruz et al., 2017). To achieve this, the authors first performed time lapse imaging and showed how cysts emerge from tubular segments and rapidly expand by partial detachment from the mutant organoids. To further prove that the loss of adherent forces was related to cyst outgrowth, organoids were cultured in suspension. The removal of adherent cues dramatically increased cyst formation rates up to 10-fold compared to standard used adherent culture systems. Importantly, cyst formation rate was assessed upon several months in culture demonstrating that CRISPR-mutant organoids showed cysts which expanded to 1-centimetre diameter compared to wild type isogenic controls. These observations recapitulated phenotypic features of early stage PKD-cysts which showed a lining epithelium with overlapped expression of proximal and distal tubular markers except for some patches with stromal cells. Furthermore, PKD-cyst showed absence of podocytes and marginal collagen deposition in line with observations in prenatal PKD-cysts. Further gene expression profiling of cyst lining cells showed overexpression of gene sets related with cell cycle progression. The authors concluded that organoid cysts result from hyperproliferative kidney tubular epithelial cells (KTECs) as previously shown in mouse and human PKD-cyst samples. Importantly, KTECs were able to proliferate and migrate as monolayer explants in the absence of stroma when kidney organoids were cultured over an ECM. Further protein expression analysis in KTECs outgrowths and undifferentiated hPSCs revealed a strong down regulation of PC1 protein expression in PC2 defective cells. Conversely, PC2 expression levels were unchanged in PC1 defective cells. Decrease in PC1 protein expression was reproduced by knocking down PC2 protein in control hPSCs. These results indicated that PC2 was required for human PC1 expression in contrast to previous studies in mice. To prove the implication of PC1 in adhesion and ECM remodelling, kidney organoids derived from PKD1 mutants and wildtype isogenic lines were embedded into collagen droplets and cultured in suspension. Only wildtype organoids were able to noticeably compress droplets in contrast to PKD1 mutants. This approach demonstrated that kidney organoid epithelia can remodel ECM microenvironment with apparent dependence on PC1. Biochemical stimuli which is characteristic of PKD were also challenged in this model system. Specifically, treatment with cAMP signalling agonists induced cyst formation in both *PKD* mutants and wildtype derived organoids. Later, the same group proved the possibility to scale up the production of CRISPR engineered organoids in a high throughput format (HTS) (Czerniecki et al., 2018). Organoids also reproduced cystic swelling response to cAMP signalling agonist treatment, demonstrating the feasibility of this format for screening applications. As a proof of concept, the authors performed a small-scale screen with eight candidate factors and an inhibitor of non-muscle myosin II (NMII as a potent inducer of cystogenesis). These results suggest on the role of polycystins maintaining cytoskeletal stability in tubules through actomyosin activation.

One major limitation of the discussed studies is the usage of protocols that differentiate towards the MM and consecutive nephron organoids. However, in patients with ADPKD, cysts are known to originate primarily from collecting duct cells which are not present in kidney organoids with mostly nephron-like structures (Devuyst et al., 1996). In this context, the Nishinkamura laboratory recently reported the use of *PKD1* mutant hiPSCs to generate UB organoids with cyst forming capacity providing a robust *in vitro* ADPKD model (Kuraoka et al., 2020). They electrocorporated a designed sgRNA vector targeting the exon 15 of *PKD*, Cas9 expression vector and targeting vector into a human iPSCS line and conducted a puromycin selection for *PKD* ^*−/−*^ clones. After differentiation of the mutant cell line and wildtype control towards nephron organoids they showed that, upon stimulation of cAMP signalling with forskolin, the *PKD* ^*−/−*^ -derived kidney organoids developed more severe cysts than the wildtype control. Interestingly, the cysts appeared mainly in LTL positive (proximal tubule) cells and WT1+ glomerular parietal epithelial cells independently of the genotype, consistent with data in mice ADPKD model (Ahrabi et al., 2010). Next the mutant *PKD* ^*−/−*^ hiPSC cell line and the wildtype control line were induced to form UB organoids. Upon treatment with forskolin, PKD mutant UB organoids developed pronounced cysts. Importantly, cysts did not develop in wildtype control UB organoids in contrast to wildtype nephron organoids that showed moderate cyst formation. In addition, they found that arginine vasopressin receptor 1A gene (AVPR1A) was functionally expressed in UB organoids. Treatment of UB organoids with the natural cAMP ligand vasopressin resulted in efficient cyst formation in *PKD* ^*−/−*^ but neither in *PKD1* ^*+/−*^ nor in wild-type UB or in nephron organoids. Moreover, they validated these findings using iPSCs derived from a patient with a heterozygous mutation in the *PKD1* gene, showing that UB organoids derived from the patient iPSCs evidenced robust cyst formation upon forskolin stimulation.

To overcome issues related to kidney organoid inter and intra batch variability Low and colleagues reported an approach in where the CRISPR/Cas9 system was used not only to model a disease but also to correct a patient-specific *PKD1* mutation in patient derived hiPSCs (Low et al., 2019). In line with previous studies, subsequent differentiation of corrected and non-corrected *PKD1* hiPSCs lines towards kidney organoids showed a low incidence of spontaneous cyst formation in patient derived kidney organoids. As in previous studies, intracellular levels of cAMP were stimulated to boost cyst incidence. After treatment, kidney organoids derived from PKD1 hiPSCs lines showed a severe increase in cystogenesis, while CRISPR-corrected isogenic controls showed marginal cyst formation. Remarkably, kidney organoids derived from PKD1 hiPSCs lines exhibited phenotypic features observed in PKD patients, such as proximal to distal enlargement of the tubule lumen, reduction in the expression of segment-specific markers, distorted glomeruli squeezed between cysts, and unfunctional proximal tubular cells at the cyst lining. Furthermore, the authors assessed the effect of two chemical compounds as blockers of cystogenesis in kidney organoids derived from PKD1 hiPSCs lines. Altogether, these results set the basis for the utilization of kidney organoids as a preclinical model for drug screening applications. In contrast with previous studies, this approach allowed for personalized studies because both mutant hiPSCs and CRISPR corrected isogenic controls comprise patient specific genetic backgrounds. In the same manner, the Freedman group also studied on the impact of the knock-out of *PODXL* in podocyte differentiation and function through the generation of kidney organoids from CRISPR gene-edited hPSCs lacking *PODXL* (*PODXL* ^*−/−*^) (Kim et al., 2017). Using Transmission Electron Microscopy (TEM) the authors showed that podocytes derived from *PODXL* ^*−/−*^organoids exhibited a drastic reduction in microvilli compared to wildtype controls. Consequently, *PODXL* defective podocytes reduced their lateral interspaces by increasing the formation of lateral cell-cell junctions. In contrast, confocal imaging of control podocytes showed extended lateral interspaces with apical microvilli expressing PODXL. Defects of human mutant podocytes *in vitro* were phenocopied in *PODXL* deficient prenatal mice. Furthermore, *Podxl* ^−/−^ mice die of anuria shortly after birth. Altogether these findings demonstrated the central role of *PODXL* in the formation of microvilli in podocytes. hPSCs allowed the characterization of PODXL mediated cell interactions at the biophysical and transcriptomic level. On one hand, when dissociated with optical tweezers, control hPSCs exhibited an anti-adhesive effect increasing cell to cell separation while *PODXL* ^*−/−*^cells remained in closer distance. This effect was explained by the presence of electrostatic repulsion forces between negative PODOCALYXIN charges presented in adjacent cell membranes of PODXL expressing cells. On the other hand, comparative transcriptomic analysis by RNA-Seq identified that *PODXL* ^−/−^ hPSCs upregulated the expression of genes related with cell adhesion concomitantly with a downregulation in the expression of genes involved in microvillus formation. Altogether, these results proposed a model in which PODOCALYXIN induces apical-to-basal junctional migration by its progressive localization at apical and lateral cell membranes of podocytes in where PODOCALYXIN induces microvilli formation. Electrostatic repulsion between apical microvilli limits cell-cell contact to the basal membrane of podocytes and generates lateral interspaces between them for proper glomerular filtration. This model predicts that loss-of-function mutations in human *PODXL* may cause embryonic or perinatal lethality due to kidney failure, as podocyte microvilli are critical for urine production in mammals. In line with this expectation, only few studies reported PODOCALYXIN mutations in living patients with kidney disease. To date only few heterozygous non-synonymous or nonsense mutations have been identified in a reduced number of patients with focal and segmental glomerulosclerosis. Remarkably, only one study reports a neonatal patient with biallelic nonsense mutations in *PODXL.* Unfortunately, the newborn early died afterbirth (∼4 months) presenting severe nephrotic syndrome and omphalocele. These features are similar to main defects described in Podxl-deficient mice. On balance, this work showed that CRISPR/Cas9 engineered organoids offer a predictive power to discover novel mechanisms explaining disease gestation and progression. A summary of recent published reports on the use of CRISPR/Cas9 technology to model renal disease is compiled in Table 2.

**TABLE 2:** Published work about disease modelling combining kidney organoids and CRISPR genome editing.

Author and year	Stem cell line	Editing method	Target genes	CRISPR induced mutations and strategy	Target disease	Differentiation protocol	Findings in mutant kidney organoids
Freedman et al. (2015)	H9 hESCs	Transfection with both Cas9-GFP expressing vector and gRNAs. Subsequent isolation of green fluorescent cells by FACS and clonal expansion for final genotyping screen by Sanger Sequencing.	PKD1 and PKD2	Biallelic loss-of- function indels.	Polycystic kidney disease (PKD)	Freedman et al. (2015)	Large, translucent, cyst-like structures emerged from tubular structures with low incidence.
Cruz et al. (2017)							Cyst lining epithelium showed overlapped expression of LTL and ECAD.
Czerniecki et al. (2018)				CRISPR-edited hESCs were used to characterize the disease related phenotype.			Cyst-lining cells were hyperproliferative, coated with primary cilia and formed tight junctions in a cobblestone pattern.
							In long-term culture, cysts accumulated a subpopulation of stromal cells and presented scant collagen deposition as prenatal clinical PKD cysts.
				Non-edited hESCs were used as isogenic wildtype controls.			Removal of stroma stimulated proliferation and migration of organoid cells in form of expanded outgrowths.
							PKD1 defective organoids were unable to contract collagen droplets through migratory forces.
							Chemical stimulation of intracellular cAMP level induced rapid, reversible and dose-dependent swelling of organoid’s tubules into cyst-like structures.
							Cysts were also produced in automated high throughput organoid cultures.
							A small-scale screen of possible PKD modulators, with organoids grown in the high throughput format, identified an inhibitor of non-muscle myosin II (NMII) as a potent inducer of cystogenesis.
Freedman et al. (2015)	H9 hESCs	Transfection with both Cas9-GFP expressing vector and gRNAs. Subsequent isolation of green fluorescent cells by FACS and clonal expansion for final genotyping screen by Sanger Sequencing.	PODXL	Biallelic loss-of- function indels.	Segmental glomerulosclerosis	Freedman et al. (2015)	Podocyte junctional markers adopted a more diffuse expression pattern.
Kim et al. (2017)							Podocytes with drastic reduction in microvilli and consequently reduced interspaces wich were enriched with lateral cell-cell junctions.
				CRISPR-edited hESCs were used to characterize the disease related phenotype.			Absence of electrostaic repulsion forces mediated by negative PODXL charges when mutant cellls were dissociated with optical tweezers.
				Non-edited hESCs were used as isogenic wildtype controls.			Upregulated expression of genes related with cell adhesion as well as downregulated expression of genes involved in microvillus formation.
Forbes et al. (2018)	hiPSCs from dermal patient fibroblasts (GM10287)	Simultaneos reprogramming and gene-editing protocol. Co-transfection of reprograming plasmids, mRNA encoding Cas9-Gem variant, sgRNA expression plasmid and a repair template plasmid. Emerging iPSCs were clonal expanded for final genotyping screen by allele specific PCR and final confirmation by Sanger Sequencing.	IFT140	Correction of the c.634G>A variant and an additional synonymous 3 bp Cas9-blocking mutation.	Hereditary ciliopathic renal disease.	Adapted from Takasato et al., 2015	Tubular epithelium showed shorter cilia with accumulation of IFT proteins swelling the ciliary tip and creating a club like shape.
				CRISPR-edited hiPSCs were used as isogenic wildtype controls.			Downregulation of genes associated with apicobasal polarity, cell-cell junctions, and dynein motor assembly.
				Non-edited hiPSCs were used to characterize the disease related phenotype.			Consistently fewer EPCAM + epithelial cells developed spheroids with polarized epithelium. Cilia per nucleus were lower in mutant spheroids.
Low et al. (2019)	hiPSCs from patient fibroblasts (GM10287)	Co-transfection of pCas9-GFP and pCAGmCherry-gRNA plasmids with the ssDNA donor oligo by electroporation. Subsequent isolation of GFP+/mCherry + cells by FACS and clonal expansion for final genotyping screen by Sanger Sequencing.	PKDH1	Correction of c.11630delT mutation.	Autosomal recessive polycystic kidney disease (ARPKD)	Low et al. (2019)	Low indicence of cysts formation by time-dependent proximal to distal enlargement of the tubule lumens.
							Chemical stimulation of intracellular cAMP level boosted cyst incidence.
				CRISPR-edited hiPSCs were used as isogenic wildtype controls.			Reduction in the expression of segment-specific markers.
							Distorted glomeruli were squeezed between cysts.
				Non-edited hiPSCs were used to characterize the disease related phenotype			Proximal tubular cells were unable to take up dextran.
							Cystic organoids responded to two already known chemical blockers of cystogenesis.
Kuraoka et al. (2020)	hiPSCs from patients (CiRA00007 and CiRA00009) hiPSCs line 201B7	Electrocorporation of iPSCs with exon15 of PKD1 targeting sgRNA, Cas9 expressing vector and targeting vector with consecutive selection with puromycin.	PKD1	CRISPR-edited cell line 201B7 as well as patient-derived cell lines were differentiated towards cyst forming nephron and UB organoids.	Autosomal polycystic kidney disease.	Adapted from Taguchi and Nishinakamura, 2017	Nephron organoids with PKD1 mutation formed more severe cysts upon forskolin treatment in comparison to controls. Cysts originated from proximal tubule and glomerulum. No response to vasopressin.
				Non-edited hiPSCs were used as controls.			Mutant UB organoids formed cysts upon forskolin as well as vaspressin tratment whereas control UB organoids did not.
Wahele 2021	hiPSC of not named origin	Infection of iPSCs harboring a doxycycline (DOX)-inducible Cas9 protein with lentiviruses driving expression of a WT1-specific guide RNA (gRNA1).	WT1	Genome editing was induced at different time points of kidney organoid differentiation by doxycyline.	Wilms Tumor Disease	Adapted from Morizane et al., 2015	Loss of WT 1 leads to hypoplasia in organoids
							WT1 coordinates epithelialization and exit from the progenitor cell state
							WT1 mutant organoids resemble human Wilms tumors with ectopic myogenesis
							Untransformed niche cells are required for long-term propagation of WT1 mutant cells

Comparison of recent works on disease modelling using kidney organoids derived from CRISPR edited human pluripotent stem cells (hPSCs) including the following information: hPSCs lines used including human induced pluripotent stem cells (hiPSCs) or human embryonic stem cells (hESCs); the method used for CRISPR mediated genome editing in hPSCs; genes targeted and the type of mutation induced as well as the role of hPSCs edited lines during phenotypic characterization of the target disease in kidney organoids; the protocol used for kidney organoid differentiation (see Table 1) and the disease specific features identified in kidney organoids derived from mutant hPSCs and isogenic wildtype controls.

The Little group demonstrated what powerful tool the combination of CRISPR/Cas9 and organoids technology can be in order to reach important perspectives of regenerative medicine as a possible therapy of genetic disorders (Forbes et al., 2018). They reprogrammed cells from a patient with nephronophtisis ciliopathy with a *IFT140* mutation to hPSCs and corrected that mutation in one step. Patient-derived cells were reprogrammed with seven transcription factors in a vector–free manner. Simultaneously *IFT140* gene was targeted by a EGFP reporter and homologous recombination facilitated by using *in vitro* transcribed mRNA encoding Cas9, a plasmid encoding a gene specific short-guide RNA and a donor template encoding EGFP reporter and a puromycin resistance gene flanked by homology arms specific to sequences upstream and downstream of the *IFT140* start codon. This protocol had been developed by the lab earlier and is supposedly highly efficient. Proband-derived iPCS as well as corrected iPSCs were differentiated to kidney organoids. The former differentiated to organoids with shorter, club-shaped cilia which is in line with findings in mouse models (Miller KA et al., 2013). Tubules showed a phenotype that is characteristic in nephronophthisis. This study did not only demonstrate the successful correction of a mutation associated with nephronophthisis but also unveiled pathogenic pathways not previously described in *IFT140*-deficient disease models. Therefore, not only differences in between species regarding disorders of the cilia was overcome which had hampered the quest to a deeper understanding. The same group went further on to target *PKHD1* gene which causes autosomal-recessive polycystic kidney disease in order to demonstrate that distal nephron epithelium that is part of the nephrons of their organoids is indeed functional. A GATA3^+^/EPCAM^+^ positive population in their organoids transcriptionally matches a distal/connecting segment the most. This population was propagated by culturing in a specific ureteric endothelium (UE) favoring medium or conditions, respectively. They exploited the established protocols to generate reporter cell lines as described earlier and targeted *PKHD1* in a GATA3 ^mCherry^ reporter cell line. CrRNAs were designed to bind within the *PKHD1* gene in order to reach a homozygous deletion and generation of a premature stop codon within the exon 4 of the *PKHD1* coding region. *PKHD1* ^*null*^ cells were differentiated, purified by FACS and propagated in UE favoring conditions. Upon transition to stalk medium containing aldosterone, vasopressin, FGF2 and retinoic acid the organoids displayed large cyst-like structures. Interestingly, these cysts could also be induced by forskolin (Howden et al., 2021). Here, disease modelling and establishment of UE culturing profit one another. Interestingly, this approach does question the paradigm that they and Morizane et al. had earlier established as in that duration of Wnt signalling will decide on lineage fate exclusively either towards MM or UB (Takasato et al., 2015 and Morizane et al., 2014).

Waehle et al. generated a (Wilms Tumor 1) *WT1* knock-out (KO) cell line by inducing hPSCS harbouring a doxycycline-inducible Cas9 protein with lentiviruses driving expression of a *WT1* specific guide RNA and a red-fluorescent protein. They induced gene editing at different time points (prior to differentiation, during nephrogenesis) following the Morizane protocol to differentiate hPSCS to organoids. *WT1* is a tumor suppressor gene and its homozygous loss is associated with Wilms Tumor, the most common kidney cancer in children. (Hendry and Little, 2012). *WT1* continues to be expressed in the NPs as well as the developing nephrons (Pelletier et al., 1991). Studying *WT1* not only is promising in leading to deeper insights in Wilms Tumor, but it is also a marker of the MM during nephrogenesis and therefore helpful to determine its function in organ development better. *WT1* KO organoids showed to be smaller compared to the wildtype which is caused by *WT1* inducing an overgrowth of NPCs at the expense of tubular/glomerular differentiation. RNA-seq revealed that NPC markers are induced and decline in control organoids while they persist in *WT1* KO organoids as well as mesenchymal to epithelial transition genes were perturbed in KO organoids. Furthermore, KO line-derived organoids transcriptionally and phenotypically resembled a subset of WT1 patients and remained in a pre-epithelialized state. Although protocols and procedures used in this study are established, the successful match and capacity of KO organoids to recapitulate phenotypic and genetic changes of *WT1* in humans despite the earlier shortcomings of the organoid technology is exciting.

## 4 Conclusions and perspectives

Taken together, the multiple procedures described for the recapitulation of early stages of kidney development using hPSCs are showing the production of complex 3D culture systems to model human kidney development and disease. These last years have shown the amenability of hPSCs for further transgenesis taking advantage of the CRISPR/Cas9 system, including targeting fluorescent reporters to identify lineage-specific cell types within the organoid, and correlate and trace these processes during differentiation. Furthermore, it is also possible to introduce fluorescent reporters under the endogenous regulation of a renal cell type-specific gene, avoiding undesired responses due to the genetic context and thus provide novel approaches to understand the embryonic origin of a desired cell type. In the next years, these approaches may shed light into some of the questions discussed in the present review, such as the developmental origin of UB cells or endothelial cells in human kidney embryogenesis compared to other model systems as mice. Other advantages of CRISPR stands on the possibility to target Cas9 into any of the well-known safe harbour *loci* of hPSCs (to date *AVVS1*, *ROSA26* or *CLYBL*) for further applications which may include the generation of multiple knock-out, the introduction of single nucleotide alterations, as well as generation of inducible knock-out during hPSC differentiation (Gonzalez et al., 2014). Such approach may also facilitate the generation of more complex genomic modifications, such as the generation of reporter alleles *via* HDR-mediated gene targeting using long donor DNA templates encoding protein tags or fluorescent proteins. On the other hand the combination of deactivated Cas9 (dCas9) fused to functional transcriptional repressors (Krüppel-associated box -KRAB) (Gilbert et al., 2013) or activators [as the tripartite activator VP64-p65-Rta (VPR) module] (Chavez et al., 2015) may open new venues for the simultaneous activation or repression of endogenous coding and noncoding sequences and thus expand our armamentarium when modelling complex renal disorders arising as secondary complications from other major complications (including hypertension, diabetes, among others).

The lessons learnt from these last years, show that there are still important drawbacks that need to be overcome when envisioning kidney organoids as faithful models to target CKD. In this regard, the lack of a vascular component and the immatureness of these tissue surrogates represent major issues that preclude the immediate application of these model systems in renal disease modelling. In this regard, technologies including scRNA-seq are helping the field to categorize the existence and maturation of the organoid cell types and to match their transcriptomic status to that found in the native tissue (including both embryonic and adult kidney counterparts). Exploiting scRNAseq has also being crucial when reference laboratories in the field of kidney development and disease (i.e., Humphreys, Little and McMahon, among others) have established quantitative comparisons between protocols, batches, and pluripotent cell lines providing important information on how to improve protocol’s reproducibility and quality (Wu et al., 2018; Phipson et al., 2019; Subramanian et al., 2019). In the same line, transcriptomic data sets have also been used to reconstruct lineage trajectories to promote maturation of desired kidney cell types or inhibit differentiation of undesired off-target cell types. In the next years the convergence of scRNAseq and kidney organoid technology is expected to provide crucial information on how to externally guide kidney differentiation in a predictable manner and further exploit these cell culture platforms to model renal disease and perform drug screening.

Hopefully, CRISPR-editing in hPSCs derived kidney organoids and further generation of renal-reporter cell lines will continue to expand our understanding on kidney development and disease. Further approaches from the bioengineering field will also increase our capacities to develop complex 3D organoids for applications in renal disease modelling. All these advances together with current efforts in the generation of new Cas9 variants with reduced off-target effects together with high-stringency criteria for sgRNA design will have a strong impact for transitioning from basic research to precise medicine application exploiting hPSC-derived kidney organoid models.

## Glossary

Chronic kidney disease: Condition characterized by a gradual loss of kidney function over time based on the presence of kidney damage or glomerular filtration rate (GFR <60 ml/min per 1.73 m2) for ≥3 months, and it is classified into five stages based on the level of GFR
Metanephros: Phase of kidney development during the fifth week of gestation in which ureteric bud start to develop into collecting duct system
Intermediate mesoderm: A region of embryonic mesoderm extending anterior–posterior between the paraxial, or somitic, mesoderm and the lateral plate mesoderm
Collecting duct system: Tubes that receive and concentrates urine from the distal convoluted tubule of nephrons and exudes it into the renal pelvis
Ureteric bud: An epithelial tube that arises from the nephric duct during embryonic development
Metanephric mesenchyme: The region of posterior intermediate mesoderm adjacent to the nephric ducts from where to the epithelial cells of the kidney emerge upon kidney induction
Cap mesenchyme: Comprised of cells which epithelialize and sequentially form the pretubular aggregate (PA), renal vesicle (RV), C-, and S-shaped bodies, and finally the mature nephron
Kidney induction: The activation of the epithelial-specific program in the metanephric mesenchyme in response to signals that promote ureteric bud invasion and branching to give rise to the renal collecting duct system
Renal vesicle: The first polarized epithelial derivative of the induced metanephric mesenchyme that is found abutting the branching tips of the ureteric bud
Nephron progenitor cells: Self-renewing cells which are able to give rise to all the cell types of the kidney. They are involved in the homeostasis and repair of the kidney and they have a therapeutic potential for treatment of kidney failure
Stroma progenitor cells: Embryonic connective tissue that is derived from the mesoderm and that differentiates into hematopoietic and connective tissue
Human pluripotent stem cells: Stem cell that has the potential to differentiate into any of the three germ layers: endoderm, mesoderm, or ectoderm
Human embryonic stem cells: Pluripotent stem cells derived from the inner cell mass of a blastocyst, an early-stage pre-implantation embryo
Human induced pluripotent stem cells: Type of pluripotent stem cell that can be generated *in vitro* from a somatic cell
Embryoid bodies: Three-dimensional aggregates of pluripotent stem cells
CRISPR/Cas9: It is a prokaryotic immune system that confers resistance to foreign genetic elements such as those present within plasmids and phages and provides a form of acquired immunity. This system has been used as gene editing technique that enables geneticists and medical researchers to edit parts of the genome by removing, adding or altering sections of the DNA sequence
Single cell RNA sequencing: Technique which provides the expression profiles of individual cells with optimized next generation sequencing technologies, giving a higher resolution of cellular differences and a better understanding of the function of the cell in its microenvironment
Reporter cell line: Engineered cell in which a specific gene is modified in its regulatory sequence in order to measure or identify the expression or being used as selectable markers
